# Stimuli-Responsive Aliphatic Polycarbonate Nanocarriers for Tumor-Targeted Drug Delivery

**DOI:** 10.3390/polym12122890

**Published:** 2020-12-02

**Authors:** Adrian Domiński, Tomasz Konieczny, Khadar Duale, Monika Krawczyk, Gabriela Pastuch-Gawołek, Piotr Kurcok

**Affiliations:** 1 Centre of Polymer and Carbon Materials, Polish Academy of Sciences, 34, M. Curie-Skłodowskiej St, 41-819 Zabrze, Poland; adrian.dominski@cmpw-pan.edu.pl (A.D.); tomasz.konieczny@cmpw-pan.edu.pl (T.K.); khadar.duale@cmpw-pan.edu.pl (K.D.); 2 Department of Organic Chemistry, Bioorganic Chemistry and Biotechnology, Faculty of Chemistry, Silesian University of Technology, Krzywoustego 4, 44-100 Gliwice, Poland; monika.krawczyk@polsl.pl (M.K.); gabriela.pastuch@polsl.pl (G.P.-G.); 3 Biotechnology Centre, Silesian University of Technology, Krzywoustego 8, 44-100 Gliwice, Poland

**Keywords:** aliphatic polycarbonates, nanocarriers, stimuli-responsive, drug delivery systems

## Abstract

Nanoparticles based on amphiphilic copolymers with tunable physicochemical properties can be used to encapsulate delicate pharmaceutics while at the same time improving their solubility, stability, pharmacokinetic properties, reducing immune surveillance, or achieving tumor-targeting ability. Those nanocarriers based on biodegradable aliphatic polycarbonates are a particularly promising platform for drug delivery due to flexibility in the design and synthesis of appropriate monomers and copolymers. Current studies in this field focus on the design and the synthesis of new effective carriers of hydrophobic drugs and their release in a controlled manner by exogenous or endogenous factors in tumor-specific regions. Reactive groups present in aliphatic carbonate copolymers, undergo a reaction under the action of a stimulus: e.g., acidic hydrolysis, oxidation, reduction, etc. leading to changes in the morphology of nanoparticles. This allows the release of the drug in a highly controlled manner and induces a desired therapeutic outcome without damaging healthy tissues. The presented review summarizes the current advances in chemistry and methods for designing stimuli-responsive nanocarriers based on aliphatic polycarbonates for controlled drug delivery.

## 1. Introduction

Nowadays, cancer is one of the biggest health problems in modern society, remaining one of the top three leading risk factors for global mortality [1]. Tumor cells proliferate uncontrollably at a much faster rate compared to normal ones. Due to this, they are characterized by many abnormalities and a combination of mutagenic stages [2]. Furthermore, tumor tissues induce resistance to growth inhibition, apoptotic mechanism, or immune surveillance [3,4,5,6], at the same time causing angiogenesis and metastases to other places in the body by interacting with the surrounding tissues [7,8]. Conventional chemotherapy, in combination with surgical resection or radiation, is currently one of the main strategies for cancer treatment in clinics. However, commonly used chemotherapeutic agents have some limitations, such as (i) poor solubility in water, (ii) high toxicity, or (iii) being rapidly metabolized and removed by the kidneys [9,10]. For example, in the case of intravenous injections of paclitaxel, almost 50% of the dose is removed from the body within the first 24 h, and less than 0.5% of the total dose is locally available to treat tumors within the lung [11]. These limitations, in combination with the growing multi-drug resistance of cancer cells to a significant number of clinical pharmaceutics [12], the limited stability of these drugs, their nonspecific toxicity, and lack of tumor-targeting ability are major obstacles to obtaining effective anticancer therapy [13,14,15,16]. Therefore, new strategies for safe and efficient anticancer therapy are urgently needed. One solution to these problems is the concept of applying nanotechnology to obtain more effective cancer treatment while minimizing the side-effects [17]. Nanocarriers have gained significant attention over the last decade and have become a promising candidate for an efficient drug delivery system for highly hydrophobic anticancer therapeutics [18,19]. Recently, various nanocarriers with different molecular architecture including liposomes [19], inorganic nanoparticles [20] or polymer-based nanoparticles such as micelles [21] or polymersomes [22] have become hot research topics in the last decade for applications in drug delivery systems (DDS). Nanoparticles can carry a drug linked to the carrier via a covalent bond, thus resulting in polymer-drug conjugates. The conjugated drug is an inactive derivative that is biotransformed in vivo to release an active drug molecule, thereby allowing drug delivery to the disease affected area and providing effective therapy [23,24]. The drug conjugation strategy minimizes the side effects before the active drug derivative reaches the molecular target [25]. However, drug conjugation involves a chemical reaction, which often changes the chemical structure, i.e., drug stereochemistry [26,27]. As a result, the biological activity of the drug is often deactivated, or drug toxicity can occur [28,29,30]. With this in mind, it seems to be a safer approach to encapsulate drugs inside nanocarriers through hydrophobic interactions. Nanoparticles can be used to encapsulate delicate pharmaceutics to improve solubility, stability, pharmacokinetic properties and a reduction in immune surveillance, or to achieve tumor-targeting ability [31]. The use of the nanoparticle strategy as a DDS is the starting point for research into the mechanisms that determine the interactions between nanoparticles and cell membranes [32]. The effect of the physicochemical properties of nanoparticles, including molecular structure [33], size, stiffness, shape, or chemical composition of the surface, on cellular uptake, has been extensively discussed [34]. In particular, nanoparticles based on biodegradable polymers are a promising platform for effective cancer treatment due to flexibility in design and synthesis. In addition, there are no issues of nanoparticle accumulation in the body because, biodegradable polymers degraded in vivo into oligomers, monomers, or low molar mass molecules, which might be removed from the body by the normal pathways [35]. Nowadays, polymeric nanocarriers based on biodegradable PEGylated aliphatic polyesters such as polylactide [36], poly(lactide-*co*-glycolide), [37], poly(ε-caprolactone) [38], bacterial or synthetic poly(3-hydroxybutyrate) [39,40] or aliphatic polycarbonates [41,42], are widely studied for the controlled drug release systems because they are all approved by the American Food and Drug Administration (FDA). The widely studied strategy for prolonging the circulation of drug carriers in the bloodstream is to coat the surface of nanoparticles with a hydrophilic polymer i.e., biocompatible polyethylene glycol (PEG) [43]. The PEGylation resulting in amphiphilic copolymer enables the self-organization of such copolymers into micelles, polymersomes, etc. featuring an outer hydrophilic PEG shell and hydrophobic polymer as the core [44]. The hydrophilic shell of the nanocarrier prevents steric recognition by the immune systems and its removal by the reticuloendothelial system from the bloodstream [45]. However, the non-biodegradability of PEG is the main obstacle to *in vivo* application. In addition, the use of PEG with a molar mass higher than 10,000 Da is problematic, as it cannot be filtered out by the human kidneys due to its large hydrodynamic radius [46]. Therefore, non-biodegradable PEGylated copolymers and free PEG accumulate in the liver with unspecified toxicological consequences [47]. Alternatively, hydrophilic polymers such as poly(vinylpyrrolidone), poly(2-methyl-2-oxazoline), poly [*N*-(2-hydroxypropyl) methacrylamide], poloxamers, chitosan, poly(*N*,*N*-dimethyl acrylamide), poly(ethyl ethylene phosphate), poly(oligo ethylene glycol methacrylate) have also been extensively studied as an outer hydrophilic shell of nanoparticles in combination with various hydrophobic (co)polymers [36,37,38,39]. Nevertheless, PEG is approved by the FDA for biomedical applications. The first FDA-approved nano-prodrug Doxil^®^ (PEGylated liposomal-doxorubicin) for clinical use in the treatment of ovarian, breast cancers, Kaposi’s sarcoma, or multiple myeloma achieved great success [48]. Therefore, PEGylated copolymers are the most widely studied in nanoparticle-based drug delivery systems. As hydrophobic copolymers for application in biomedical fields aliphatic polycarbonates (APC) deserve special attention due to their excellent biocompatibility, biodegradability, non-toxic degradation products, lack of autocatalytic degradation, and broad-tuned functionality to achieve efficient tumor-targeting performance [49,50]. The synthetic approaches towards aliphatic polycarbonates consist of three major polymerization methods [51,52]: (i) polycondensation of carbonates and diols, (ii) copolymerization of epoxides with carbon dioxide, and (iii) ring-opening polymerization (ROP) of cyclic carbonates. Significant advances in ROP of cyclic carbonates initiated with metal-free basic organocatalysts such as amidine (DBU), guanidine (TBD) or phosphazene (BEMP), organic acids i.e., methanesulfonic acid, triflic acid, diphenyl phosphate have been achieved. but also, the enzymes mediated synthesis of ACPs showed the absence of any toxic compounds, good control of the polymerization process and high reaction yields [53]. Furthermore, the synthesis of cyclic carbonate monomers utilizing 2,2-bis(hydroxymethyl)propionic acid, pentaerythritol, or glycerol as a scaffold provides an enormous amount of possibilities for APC functionalization to achieve desirable properties for biomedical applications. Due to this, the aliphatic polycarbonates have great potential as “smart” anticancer drug delivery systems with desired features such as biodegradability, non-toxicity, and stimuli responsiveness to achieve tumor-targeting ability.

Recently, many studies have focused on the design and synthesis of novel efficient drug delivery systems, designed for transporting the anticancer therapeutics directly to cancer tissues and releasing the drug in a controlled manner by endogenous or exogenous factors [54,55,56]. To develop such stimuli-responsive polymeric nanocarriers, various stimuli-labile groups are used. Due to incorporated stimuli-sensitive chemical groups into the polymeric microstructure, nanoparticles can respond to exogenous (e.g., light, electric or magnetic fields, ultrasounds) or endogenous stimulus (e.g., pH, enzymes, temperature) by disassembling, swelling, changing nanoparticle size, shape or charge shifting [57,58,59] which leads to release of the therapeutics in a controlled manner in pathologically changed place and improving the efficacy of the chemotherapy. The microenvironment of the tumor tissue differs considerably from healthy tissues. Compared to normal tissues, the cancer ones are characterized by unique pathophysiological markers, i.e., lower pH in the internal and external microenvironment, high intracellular glutathione level, a higher level of reactive oxygen species, reducing and hypoxia conditions and various specific enzyme overexpression [60,61,62], which can act as an endogenous drug release trigger to induce a desired therapeutic outcome without damaging healthy tissues. 

In fact, some of the stimuli-responsive nanocarriers based on APCs already show great potential for providing efficient drug administration with reduced side effects. In this review, we summarize recent advances in the development of stimuli-responsive nanoparticles based on aliphatic polycarbonates, highlighting the versatility of PEGylated APC in the fabrication of “smart” drug delivery systems. The synthesis and the self-assembly properties of amphiphilic APC copolymers containing various stimuli-responsive functional groups are presented. In particular, the mechanisms of drug release in the response to endogenous or exogenous factors are fully elucidated. Understanding these mechanisms might help in designing more efficient stimuli-responsive drug delivery systems.

## 2. Application of Endogenous/Exogenous Stimulus-Responsive APC Nanocarriers 

In designing new nanocarriers for anticancer drug delivery, it is vital to know and take advantage of the differences between tumor cells and healthy tissues. As mentioned earlier, the tumor microenvironment differs significantly from normal tissues, which can act as an endogenous stimulus for drug release at the desired sites of action, increasing therapeutic effect. However, tumor tissues are heterogeneous and the overexpression of various unique markers differs from cell to cell. Therefore, the use of nanocarriers sensitive to an external stimulus, due to their accuracy and non-invasiveness, so far, are more promising for obtaining an effective anticancer therapy [54].

### 2.1. pH-Responsive APC Nanocarriers

**Figure 1:**
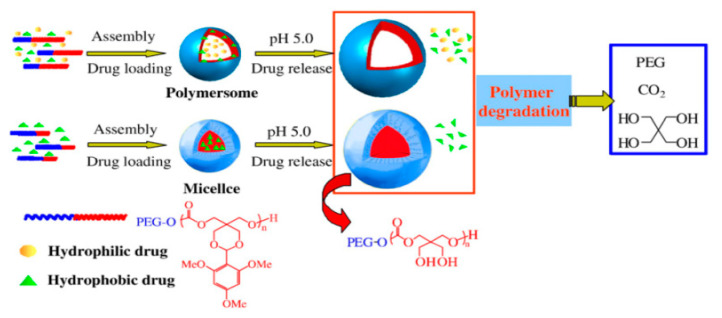
Schematic illustration of pH-sensitive degradable polymersomes or micelles based on poly((ethylene glycol)-*b*-(2,4,6-trimethoxybenzylidenepentaerythritol carbonate)) for triggered anticancer drugs delivery. Reprinted with permission from ref [70]. Copyright 2009 Elsevier B.V.

The pH gradient in the internal and external tumor microenvironment is caused by the unique metabolism of sugar derivatives by tumor tissues. Tumors consume an enormous amount of glucose compared to healthy cells, due to a fast rate of glycolysis, to provide the energy needed to increase proliferation [63]. Such a high glycolysis rate leads to the accumulated pyruvate is transformed mainly in lactic acid fermentation, which is characteristic of hypoxic conditions [64]. The relationship between the high glucose uptake and increased proliferation of cancer cells and acidification of the tumor microenvironment caused by lactate production is known as the Warburg effect [65]. It is a result of tumor’s mitochondrial metabolic abnormalities [66]. The increased glucose uptake by tumor tissues is related to the overexpression of glucose transporters, which are specific transmembrane proteins that facilitate glucose and galactose uptake within cancer cells [67]. Understanding the Warburg effect led to the development of a cancer imaging technique (positron emission tomography—PET), which tracks the radioactively labeled glucose derivate, 2-deoxy-2-[^18^F]fluoro-d-glucose, and is extremely useful for diagnosis, staging and monitoring treatment of cancers [68]. Furthermore, it is a starting point for developing tumor-targeting pH-responsive nanocarriers. In the first attempt to design and synthesize pH-responsive nanocarriers based on APC Zhong and coworkers [69] developed biodegradable pH-sensitive micelles based on a diblock copolymer of PEG and acetal-functionalized aliphatic polycarbonate (poly(2,4,6-trimethoxybenzylidene-pentaerythritol carbonate)). Amphiphilic copolymer formed micelles in an aqueous solution with an average size of about 120 nm. The pH-sensitivity of this system is caused by the presence of an acid-labile acetal bond, which is relatively stable in a slightly basic environment (pH 7.4), while it is rapidly hydrolyzed in an acidic environment (pH 5.0). The acetal group hydrolysis leads to the formation of two hydroxyl groups in the core of the micelles and, as a result, the micelles swell and release the encapsulated drug. The in vitro release studies of anticancer drugs (paclitaxel or doxorubicin) showed that the drug is released slowly at physiological pH (7.4) and much faster at endosomal pH (5.0), due to rapid acetal hydrolysis in an acidic environment. The same group also reported the preparation of polymersomes using the above mentioned PEGylated APC with the acetal groups [70]. Polymersomes ranged from 100–200 nm and the mechanism of drug release was the same as for micelles. Interestingly, in an acidic environment, polymersomes were able to release anticancer drugs faster compared to the micelles. However, polymersomes were able to simultaneously load two anticancer drugs (PTX and DOX). As a result, such a system can be used successfully in combination therapy (Figure 1). 

Very recently, Domiński et al. [71] developed pH-responsive nanocarriers consisting of poly(ethylene glycol)-acetal-functionalized APC-oligo[R]-3-hydroxybutyrate triblock copolymer micelles loaded with 8-hydroxyquinoline glycoconjugates for Warburg effect based tumor targeting. Remarkably, glycoconjugates-loaded micelles showed a significantly increased ability to inhibit the proliferation of cancer cells compared to free glycoconjugates. This is due to an enhanced uptake and pH-triggered release of glycoconjugates in the tumor microenvironment, while free glycoconjugates only showed passive diffusion through the lipid barriers of cancer cells. Glycoconjugate-loaded micelles selectively kill cancer cells (MCF-7 and HCT-116) and reduce the damage of the healthy cells (NHDF-Neo). The combination of drug modification with the stimuli-responsive nanocarrier to achieve joint action might open up novel strategies for efficient tumor therapy. 

**Figure 2:**
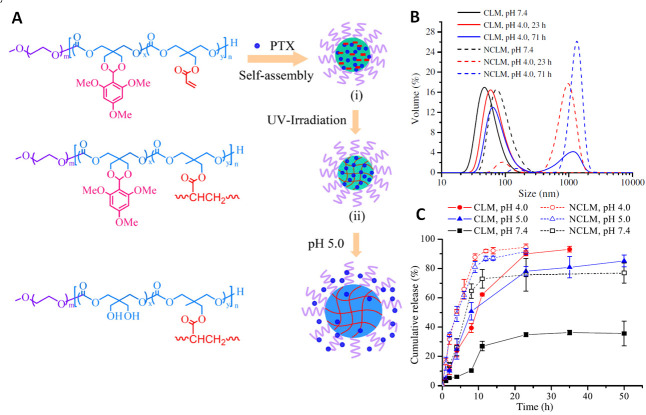
(**A**) Schematic illustration of photo-crosslinkable pH-responsive micelles based on poly(ethylene glycol)-poly(2,4,6-trimethoxybenzylidene-pentaerythritol carbonate-*co*-acryloyl carbonate) diblock copolymer. PTX-loaded crosslinked micelles exhibit superior extracellular stability while “actively” release PTX under a mildly acidic condition mimicking that of the endo/lysosomal compartments. (**B**) Size change of pH-sensitive crosslinked (CLM) and non-crosslinked (NCLM) micelles at various pH and 37 °C determined by dynamic light scattering measurements (DLS). (**C**) pH-dependent drug release from PTX-loaded crosslinked and non-crosslinked micelles at 37 °C. Reprinted with permission from ref [73]. Copyright 2012 Elsevier B.V.

Dove and coworkers developed PEGylated poly((2-norbornene-5,5-bis(hydroxymethyl) trimethylene carbonate) for pH-triggered drug release with norbornene group for versatile post-polymerization modifications [72]. Norbornene groups were easily functionalized with benzyl azide via 1,3-dipolar cycloaddition (“click” chemistry), dodecanethiol *via* photoinduced radical thiol-ene addition, and tetrazine *via* inverse electron demand Diels–Alder reaction. The other advantage of the prepared system was the incorporation of the acetal group for the pH-triggered release of a drug functionalized with norbornene molecule. The ability to accurately control the amount of drug or imaging agents grafted on the polymer backbone provides many possibilities for applications in biomedical fields. The acetal group is commonly used in pH-responsive anticancer drug delivery systems due to its rapid hydrolysis in the acidic tumor environment. However, obtaining stable nanoparticles with acetal groups is not an easy task. The acetal groups hydrolyze with the formation of two hydroxyl groups in the polymer chain, which significantly increases the hydrophilicity of the hydrophobic core of nanoparticles. It disturbs hydrophilic/hydrophobic balance, which is a crucial parameter for the amphiphilic copolymers to self-assemble into nanoparticles. Hence, Zhong et al. [73] develop core-crosslinked pH-responsive micelles to improve stability and prolong circulation time. The PEGylated poly(2,4,6-trimethoxybenzylidene-pentaerythritol carbonate-*co*-acryloyl carbonate) copolymer containing an acetal group for pH-triggered drug release and photo-crosslinkable acryloyl groups was synthesized. The micelles formed using such a copolymer were photo-crosslinked, resulting in extremely stable nanoparticles at physiological pH, while at an acidic pH they were able to hydrolyze resulting in the release of drugs. *In vitro* drug release studies confirmed that crosslinking does not influence drug release at acidic pH (4.0 and 5.0), while increased stability was observed at pH 7.4. In addition, *in vitro* cellular studies showed that cross-linked and non-cross-linked micelles exhibited similar anti-tumor activity, which may indicate that the micelles core photo-crosslinking does not affect drug release (Figure 2). 

**Figure 3:**
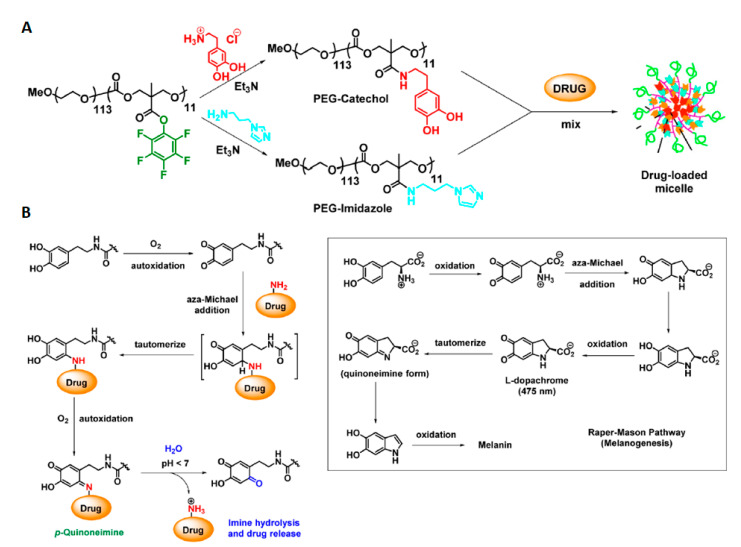
Schematic illustration of preparing (**A**) mixed micelle system based on PEGylated polycarbonate copolymers; organocatalytic anticancer drug loading. (**B**) Biomimetic mechanism of drug-loading onto the catechol side chains of the block copolymer. (inset) Raper−Mason mechanism of melanin biosynthesis. Reprinted with permission from ref [85]. Copyright 2016 American Chemical Society.

Chemical crosslinking via thiol-acrylate Michael addition reaction [74] or oxidative self-crosslinking of dopamine-grafted APC block [75] are also an effective method to obtain stable core-crosslinked micelles. Recently, to increase bioavailability and give active tumor-targeting properties, PEGylated acetal and acryloyl functionalized APC copolymer was mixed with another amphiphilic copolymer, i.e., galactose-PEG-*b*-poly(ε-caprolactone). Both amphiphilic copolymers form in aqueous solutions a type of mixed micelles with an average size around 100 nm and then are photo crosslinked utilizing the strategy mentioned above. The in vitro and in vivo studies revealed that galactose-decorated core-crosslinked pH-responsive micelles have excellent stability, biocompatibility, and are actively targeting the hepatoma cells due to receptor-mediated mechanism [76]. Cross-linking using disulfide bonding is another approach to achieving stable micelles. Zhong and coworkers synthesized PEGylated poly(2,4,6-trimethoxybenzylidene-pentaerythritol carbonate-*co*-pyridyl disulfide carbonate) a copolymer that formed approximately 60 nm micelles in aqueous solutions [77]. The micelles obtained in this way were cross-linked by the addition of dithiothreitol, which in the first step reducing the disulfide bond. Then, free thiol groups inside the micelle core were oxidized by potassium persulfate to form the disulfide bond with simultaneous cross-linking of the micelle core. In vitro drug release studies showed that such micelles were stable at pH 7.4, and less than 20% of the encapsulated drug was released within 24 h. In contrast, the drug was released much more rapidly at pH 5.0 which was caused by the hydrolysis of acetal groups. The addition of glutathione, which elevated levels are characteristic for cancer cells (vide infra), significantly accelerates the drug release, as a result of de-crosslinking of micelles. Very recently, Zhong’s group developed A6 peptide-tagged micelles with core-disulfide-crosslinked for delivery of proteasome inhibitor (carfilzomib) for targeted therapy of CD44-overexpressing LP-1 human multiple myeloma. A6 peptide (with the sequence: KPSSPPEE)-tagged core-crosslinked micelles possess excellent stability [78], glutathione-triggered drug release and CD44-targeting ability. In vivo studies revealed an increased tumor accumulation and reduction of systemic cytotoxicity compared with clinically used carfilzomib-sulfobutylether-β-cyclodextrin complex. Another approach to provide stable micelles *via* core-crosslinking was developed by Huang et al. [79,80]. The authors designed and obtained stable micelles in an interesting way using the complementarity of the base-pairing interactions forming DNA double helix. The PEGylated copolymers of poly(lactide-*co*-nucleobase-grafted-APC) (adenine or thymine respectively) were mixed to form micelles with an average size ~100 nm. The base-pairing interactions between nucleobases localized in hydrophobic micelles core caused core-crosslinking due to multiple hydrogen bonds between adenine and thymine, as occurs in the DNA double helix structure. The core-crosslinking significantly reduces the possibility of micelles reorganizing. Therefore, core-crosslinked micelles have greater stability and a lower critical micelle concentration value. The nucleobase core-crosslinked micelles were stable at pH 7.4 leading to a significant decrease in drug release. However, in an acidic environment (pH 5.0) the drug was released much faster. It is caused by the dissociation of hydrogen bonds between nucleobases in the acidic environment. He et al. [81] developed a thymine-functionalized six-membered cyclic carbonate monomer, which can be easily polymerized via enzymatic ROP in a controlled manner. The presence of thymine in the amphiphilic copolymer chain allows obtaining nanoparticles with a high-efficiency of loading with anticancer drugs e.g., methotrexate (MTX). This is because the exposed thymine group in hydrophobic micelles core form multiple hydrogen-bonding interactions with MTX molecules. Very recently, the same group designed and synthesized a PEGylated dual-functional APC copolymer possessing thymine and carboxyl groups for co-delivery of multiple drugs (MTX and DOX) using a single nanocarrier [82]. Thymine groups form a hydrogen bond with MTX, while carboxylic acid groups form electrostatic interactions with amine groups of DOX. Thereby, a single carrier can contribute to the simultaneous delivery of two different anticancer drugs for combination therapy. In a slightly acidic tumor environment, the protonation of carboxyl groups in copolymer and amino groups in doxorubicin/methotrexate reduces both hydrogen-bonding and electrostatic interactions that accelerate the release of drugs. Preliminary in vitro cellular studies confirm that such a system has the potential in clinical application for combination chemotherapy, which currently appears to be the most promising way to achieve efficient cancer treatment. Amphiphilic carboxylic acid-functionalized APC to enhance DOX loading efficiency and provide the positively charged shell of micelles were exploited by He et al. [83]. The micelles from carboxyl-modified PEGylated APC were compared with the micelles made of the unmodified copolymer. The carboxyl groups formed electrostatic interactions with amine groups from doxorubicin. Whereby, much higher drug loading efficiency and drug loading capacity of micelles were observed. Additionally, PEGylated carboxyl-functionalized APC showed a lack of initial burst drug release and a more prolonged drug release profile. Wang et al. [84] designed a smart drug delivery system based on two different copolymers to obtain pH-responsive mixed micelles. PEGylated carboxylic acid-grafted carbonate copolymer was mixed with dimethylamine functionalized polycarbonate. The dimethylamine moiety was designed as a pH-responsive trigger that causes the disintegration of the mixed micelles, resulting in an encapsulated doxorubicin release. Recently, Hedrick and coworkers developed a pH-responsive nanocarrier with a biomimetic doxorubicin conjugation mechanism [85]. Catechol-functionalized APC can form covalent DOX conjugates via a pH-sensitive *p*-quinoneimine bond by a mechanism that mimics the Raper-Mason pathway of melanogenesis. Interestingly, the addition of PEGylated *N*-methylimidazole-functionalized APC to catechol-functionalized APC during DOX conjugation with concomitant micelles formation results in higher drug loading efficiency (Figure 3). This indicates that *N*-methylimidazole behaves as an organocatalyst for such DOX conjugation. In vivo studies showed excellent biocompatibility, tumor-targeting ability, and pH-triggered drug release in tumor tissues. 

Organoboron functionalized copolymers offer broad possibilities to design pH-responsive nanocarriers due to the ability to form a reversible boronate ester bond between diols and catechol-containing molecules. Herrera-Alonso and coworkers designed and developed PEGylated phenylboronic acid-functionalized APC for the pH-triggered release of a diol containing anticancer drug—capecitabine [86,87]. The drug is conjugated to the copolymer through a pH-sensitive boronate ester bond. This approach allowed to load anticancer drugs with high loading levels, which release is accelerated at acidic pH demonstrating the utility of phenylboronic acid-functionalized nanoparticles as a promising platform for drug delivery applications. Yang and coworkers [88] also exploited the formation of boronate ester linkage between drug and polymer. To take advantage of the acidic tumor environment, they designed dual pH-responsive shell-cleavable micelles for anticancer drug delivery. Micelles consisted of poly(ethylene glycol) and catechol-containing APC with acetal bond as the linker between hydrophilic and hydrophobic copolymer parts. Whereby, obtained micelles shed the PEG “shell” at acidic pH accelerating the drug release. The catechol groups in the copolymer backbone form a boronate ester bond with bortezomib, an anticancer drug containing a phenylboronic acid group in structure. Dual pH-sensitive micelles showed much better anti-tumor activity in human breast cancer BT-474 xenograft mouse model than free bortezomib while mitigating hepatotoxicity of the drug. The same group developed PEGylated APC copolymer containing phenylboronic acid and tertiary amine groups for conjugation of apomorphine (a drug containing a catechol group), which is used for the treatment of Parkinson’s disease. The tertiary amine groups in the copolymer backbone increase conjugation efficiency. In vivo studies revealed that the intranasal administration of such nanoparticles transports them across the blood-brain barrier, which makes them a potential platform for the treatment of Parkinson’s disease [89]. 

The hydrazone bond is also an attractive linkage used in pH-responsive drug delivery systems due to its unique characteristic. At physiological pH 7.4, the hydrazone group is relatively stable and hydrolysis occurs slowly. However, in a mildly acidic environment, the rate of hydrolysis increases significantly. Especially, the hydrazone bond is a commonly used linkage for conjugation of doxorubicin through its ketone group [90]. Yang et al. [91] developed pH-responsive PEGylated APC with doxorubicin conjugated via Schiff-base linkage to circumvent multidrug resistance. The resulting amphiphilic copolymer formed stable micelles in a physiological environment with an average size of about 100 nm and a pH-dependent drug release mode. In vitro cell studies have shown that for MCF-7 breast cancer cells, the cellular uptake of free doxorubicin was higher compared to DOX-conjugated micelles. However, in the case of DOX-resistant MCF-7/ADR cells, the cellular uptake of free DOX was negligible, while DOX-conjugated micelles easily entered the cancer cells leading to effective inhibition of proliferation. Similarly, conjugation of doxorubicin via a hydrazone bond to aliphatic carbonates was developed by Ji et al. [92], using the biodegradable polymer prodrug poly(5-methyl-5-allyloxycarbonyl-1,3-dioxan-2-one)-*g*-12-acryloyloxy dodecyl phosphorylcholine-*co*-6-maleimidocaproyl-doxorubicin. He et al. [93] also grafted DOX on amphiphilic copolycarbonate. In both cases, DOX was released at mildly acidic conditions *via* the cleavage of the hydrazone bonds. Extended cellular uptake studies revealed that in both cases, micelles easily penetrated cancer cells and release DOX into the cancer cells nuclei. 

Recently, Shunmugam et al. [94] synthesized an active tumor-targeting nanocarrier based on biotin-tagged PEGylated copolymer of lactide and cyclic carbonate functionalized with propargyl groups. DOX-azide derivative containing oxime linkage has been conjugated to a copolymer through “click” chemistry. Biotin has been attached to the outer shell of micelles to ensure active tumor-targeting due to the overexpression of biotin receptors in tumor tissues. The pH-sensitivity of the system is caused by the presence of an oxime bond, which at acid pH (5.0) hydrolyses quickly releasing DOX. In vitro drug release revealed pH-dependency, while cell viability studies showed that micelles are highly effective in inhibiting cancer cell growth. 

Lately, Kuckling and coworkers developed a versatile platform for designing and synthesizing novel pH-responsive nanocarriers based on diblock copolymer poly(dimethylacrylamide)-*b*-poly(trimethylene carbonate) with imine linkage between hydrophilic and hydrophobic blocks [95]. The copolymer was synthesized *via* combined ROP and RAFT polymerization techniques which gives a huge amount of possibilities to functionalize such copolymers to achieve superior tumor-targeting efficiency. Such an approach to develop the pH-cleavable bond between hydrophilic and hydrophobic polymeric chains might expand the range of applications of aliphatic polycarbonates as tumor-targeting drug delivery systems. 

The possibility of using nanocarriers containing ionizable groups in copolymer structure for pH-triggered drug release was also studied. When these ionizable groups become protonated below the acid dissociation constant, the nanoparticles are destabilized by the charge repulsion causing reorganization and consequently, the release of the encapsulated cargo. Venkataraman et al. [96] developed an APC copolymer containing amines and zwitterions in the copolymer backbone as a versatile platform for pH-responsive vehicles for biomedical applications. Biodegradable polymers were synthesized via controlled ROP of *N*-substituted 8-membered cyclic carbonates using organocatalyst or through the combination of ROP and post-polymerization modification. Feng et al. [97] developed a random copolymer of ε-caprolactone and 16-membered cyclic dicarbonate with tertiary amine groups in the backbone via lipase-catalyzed ROP. This copolymer was fully biodegradable and biocompatible, as confirmed by in vitro studies. In addition, the higher the carbonate content in the copolymer, the faster the enzymatic degradation took place, with the formation of non-toxic degradation products. To demonstrate the potential use as a drug carrier, microspheres were obtained and loaded with ibuprofen and doxorubicin as model drugs. The pH-sensitivity of the obtained particles results from the presence of tertiary amine groups in the copolymer structure. Amine groups accept a proton, which increases hydrophilicity and releases the encapsulated drug in a pH-dependent manner. The same group further extended researches using APCs with tertiary amine groups to develop nanoparticles for controlled drug delivery systems. For example, in the form of an amphiphilic triblock copolymer ABA obtained using tertiary amine-functionalized APC diol as a telechelic initiator of enzymatic ROP of ε-caprolactone [98]. Furthermore, copolymer consisted of PEG and poly(tertiary amine-functionalized carbonate-*co*-ε-caprolactone) blocks [99], or amphoteric aliphatic copolycarbonates with amine and carboxyl groups in copolymer structure [100] were also reported. 

**Figure 4:**
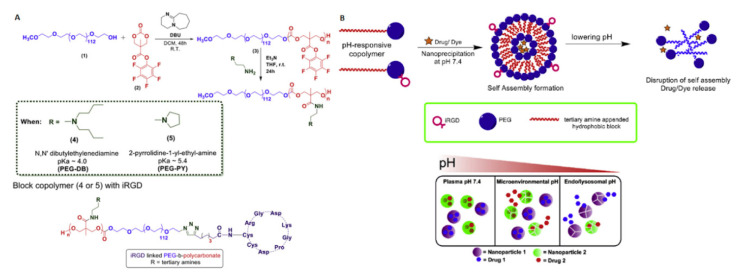
(**A**) Synthetic route to pH-responsive PEGylated polycarbonate copolymers and their iRGD conjugated variant. (**B**) (**Top**) Schematic illustration of self-assembly of pH-responsive copolymers and (**bottom**) mixed micelle system for pH-selective drugs release. Reprinted with permission from ref [102]. Copyright 2018 Elsevier B.V.

Recently, the same authors have developed micelles based on amine-functionalized copolymer, poly(6,14-dimethyl-1,3,9,11-tetraoxa-6,14-diaza-cyclohexadecane-2,10-dione)-*b*-(1,3-dioxepan-2-one) The micelles obtained had a size of ~165 nm. However, at pH 5.8, which is simulating the tumor microenvironment, the tertiary amines are ionized, leading to the micelles swelling and the encapsulated drug release. Extended studies using confocal laser scanning microscopy (CLSM) have shown an enhanced cellular internalization of camptothecin or doxorubicin-loaded micelles by cancer cells [101]. Quadir et al. [102] prepared pH-responsive iRGD peptide decorated nanoparticles consisting of PEGylated APC for pancreatic cancer combination therapy. The iRGD-peptide were immobilized on a nanoparticle shell to augment cellular uptake. The APC blocks have been functionalized with tertiary amines, such as *N*,*N*′-dibutylethylenediamine (pKa = 4.0) and 2-pyrrolidin-1-yl-ethyl-amine (pKa = 5.4) to obtain responsivity at different pH. The amines promote the disintegration of micelles in acidic pH and release of the encapsulated anticancer agents gemcitabine and a Hedgehog inhibitor (GDC 0449) in the extracellular tumor microenvironment (pH 6.0–7.0) and intracellular compartments (pH 5.5–4.5) of tumor tissues, respectively. It was also reported that a stoichiometric mixture of micelles formed from two types of pH-sensitive copolymers, enabled to achieve drug release depending on corresponding extra- and intracellular tumor microenvironment. This system was found to inhibit the proliferation of pancreatic cancer cells and showed selective internalization of nanoparticles in pancreatic tumor tissues (Figure 4).

The acidic extracellular and intracellular microenvironment of tumor tissues led to a comprehensive study of pH-triggered nanocarriers for controlled anticancer drug release. The pH-triggered drug administration is considered to be the most basic strategy to target tumor cells. Despite the many advantages of such nanocarriers, limited stability is one of the major issues. Nanocarriers must be stable before entering the cancer cell to prevent drug leakage. Obtaining stable nanoparticles is difficult due to the dynamic process of their formation and reorganization. Therefore, the combination of pH-triggered drug release with cross-linking of the micelle core (physically, chemically, or stimuli-responsive) seems to be a promising strategy to improve stability against dilution and prolonged circulation. Moreover, to enhance internalization by tumor tissues, targeting ligands that can differentiate tumor tissues from normal ones might be added. In the future, the combination of pH-responsive copolymers with other stimuli and/or the addition of active targeting ligands to nanoparticle surfaces might mitigate some of the challenges in chemotherapy.

### 2.2. Redox-Responsive APC Nanocarriers

The differences between intracellular and extracellular levels of reducing agents are broadly exploited as an endogenous stimulus for the controlled release of genes and drugs [103]. The reduced glutathione (GSH) i.e., γ-glutamyl-cysteinyl-glycine, is the most common biological reducing agent next to ascorbate, cysteine, albumin, tocopherol, β-carotene, etc. [104]. The concentration of intracellular GSH is specific for different cell types and ranges from ~2 to 10 mM while an extracellular concentration is in the range of ~2–20 μM [105]. It is known that cancer cells have at least a fourfold higher concentration of GSH than healthy cells. Carcinogenesis studies have shown a relationship between the concentration of GSH in tumor tissues and their resistance to radio- and chemotherapy. Recent studies emphasize the protective role of GSH in the process of cell apoptosis, so tumor tissues with higher amounts of glutathione are resistant to apoptosis [106]. Therefore, a decrease in GSH concentration in tumor tissues may increase the effectiveness of radio- and chemotherapy, but also stimulate rapid apoptosis of cancer cells [107]. The depletion of GSH levels causes apoptosis in pancreatic cancer cells [108], hepatoma cells [109], or B16 Melanoma cancer cells [110]. The high glutathione concentration in tumor tissues constitutes a great endogenous stimulus in designing tumor-targeting nanocarriers for the intracellular release of anticancer drugs. The disulfide bond is the most common redox-responsive linker to provide GSH-sensitive nanocarriers. It is due to the stability of the disulfide bond at physiological pH and susceptibility to reduce at a high concentration of GSH in the intracellular tumor tissues. The disulfide bond is cleaved into two thiols groups and the reduced glutathione is transformed into oxidized glutathione form, causing the rupture of nanoformulations and initiating drug release [111]. This can be achieved by designing nanocarriers containing redox-sensitive groups within the hydrophobic copolymer backbone. Li et al. [112] developed a reduction-responsive polymeric prodrug from PEGylated APC functionalized with a propargyl group grafted with N_3_-SS-Paclitaxel via azide-alkyne click reaction. The paclitaxel-conjugated amphiphilic copolymer was used to encapsulate doxorubicin to provide combination chemotherapy. To enhance stability and on-demand drug release, the nanoparticles were crosslinked by a redox-sensitive linker. The excess of propargyl groups was crosslinked by azide-alkyne click reaction with bis(azidoethyl)disulfide. Both drugs were released slowly from the nanocarriers at physiological pH, however, were significantly accelerated in the presence of intracellular reducing agent concentration (10 mM dithiothreitol). The in vitro studies conducted on HeLa and MCF-7/ADR cells revealed that the co-delivery of two anticancer drugs exhibited a synergistic effect for inhibiting the proliferation of cancer cells. Chen et al. [113] developed glyco-nanoparticles with GSH-responsive sheddable saccharide (lactobionic acid) shells for hepatoma-targeting delivery of doxorubicin. Lactobionic acid is an active tumor-targeting moiety taking advantage of hepatocellular carcinoma cells that overexpress asialoglycoprotein receptors. The lactobionic acid decorated nanoparticles were obtained from ε-caprolactone and pyridyl disulfide-functionalized APC followed by post-polymerization modification with thiolated lactobionic acid by thiol-disulfide exchange reaction. This design allows the targeting of liver cancer cells and release of DOX in the reductive tumor microenvironment by rapid shell-shedding. Flow cytometry results showed enhancement in the association of lactobionic acid decorated nanoparticles over the non-targeted nanoparticles. A similar tumor-targeting ligand is a cRGD peptide due to its binding capacity of the α_v_β_3_ integrin which overexpression occurs in most tumor tissues. That is why it has been extensively studied for tumor-targeting in the treatment of cancer [114]. Zhong and coworkers [115] synthesized a cRGD-decorated PEGylated poly(trimethylene carbonate-*co*-dithiolane trimethylene carbonate). The micelles with an average size of ~150 nm formed from this amphiphilic copolymer were loaded with doxorubicin. The micelle cores were crosslinked via a disulfide bond to minimize drug leakage and improve stability. The *in vivo* biodistribution investigation showed better therapeutic outcomes and enhanced accumulation of cRGD peptide decorated nanoparticles in tumor tissues compared to clinically used PEGylated liposomal doxorubicin. Recently, the same group has improved the cRGD-decorated nanoparticles by conjugating the anticancer drug (mertansine) via a disulfide bond to impart redox-sensitivity to drug release. Whereas, excess of thiol groups in the copolymer structure was used to crosslink micelle cores to enhance stability. The confocal microscopy studies have shown active tumor-targeting by cRGD-decorated nanoparticles to α_v_β_3_ integrin overexpressing melanoma cells. In vivo experiments revealed a significant tumor growth inhibition with cRGD-functionalized nanoparticles loaded with mertansine when compared with non-targeting nanoparticles loaded with drug and free mertansine [116]. Another well-known receptor-mediated tumor-targeting moiety is folic acid. Tumor tissues have been shown to be overexpressing folate receptors on the cell surface compared to healthy tissues [117]. This folate overexpression of tumor tissues was exploited by Lv et al. [118] to design smart folate-conjugated PEGylated APC. The micelles were crosslinked via a disulfide bond to obtain redox-responsiveness. The results of in vitro studies and confocal laser scanning microscopy revealed that the conjugation of folic acid at the surface of micelles enhanced the cellular uptake due to folate receptor-mediated endocytosis. 

**Figure 5:**
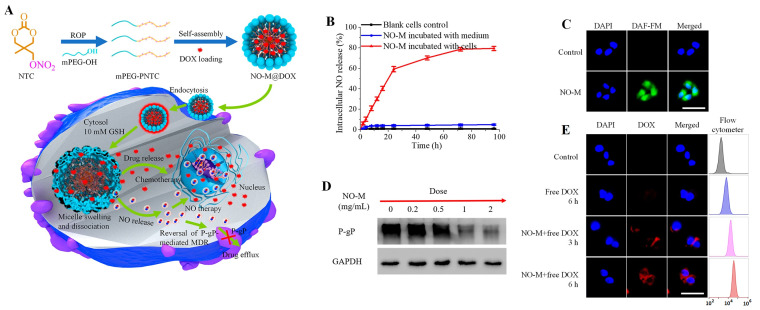
(**A**) Schematic illustration of the synthesis of nitric oxide donor-containing polycarbonate-based micelles (NO-M) for reduction-triggered drug delivery. (**B**) Intracellular NO release from NO-M in MCF7/DOX resistant cells determined by Griess reagent treatment and observed by fluorescence microscopy (**C**). (**D**) Detection of P-gP expression in MCF7/DOX resistant cells incubated with nitric oxide donor-containing aliphatic polycarbonates (APC)-based micelles at different concentrations by Western blot assay. (**E**) Fluorescence images and flow cytometer analysis for cellular uptake of free DOX in MCF7/DOX resistant cells with and without 24 h pretreatment of nitric oxide donor-containing micelles (1.0 mg/mL). The scale bars represent 50 μm. Reprinted with permission from ref [119]. Copyright 2019 American Chemical Society.

The emerging therapeutic potential of nitric oxide in cancer treatment has made the design of nanoparticles for co-delivering nitric oxide and anticancer drugs a hot research topic in recent years. To overcome multi-drug resistance, Chen and coworkers have designed reduction-triggered micelles for co-delivery of nitric oxide and doxorubicin [119]. The micelles were obtained by self-assembly of amphiphilic nitrate-functionalized APC to improve the stability of nitric oxide-donor, while triggered nitric oxide and DOX at tumor reductive conditions. In vivo studies revealed that the nitric oxide release results in P-glycoprotein inhibition to avoid multi-drug resistance and significantly enhance the DOX accumulation in tumor cells (Figure 5) to obtain an efficient therapeutic outcome.

**Figure 6:**
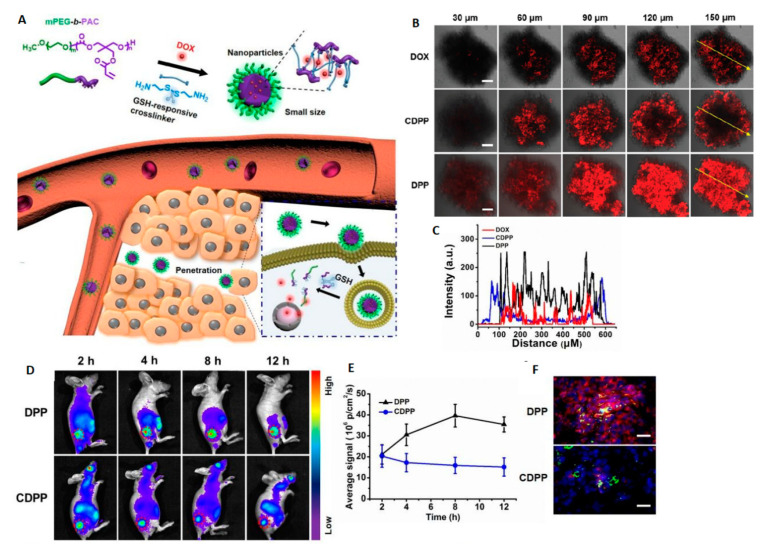
(**A**) Schematic illustration of small-sized crosslinked copolymeric nanoparticles for deep tumor penetration and intracellular reduction-triggered drug release. (**B**) Confocal laser scanning microscopy (CLSM) images of multicellular tumor spheroids treated with DOX, micelles with average size ~50 nm (CDPP), and small-sized micelles with average size ~19 nm (DPP). Scale bar represents 100 μm. (**C**) Semiquantitative fluorescence along the line drawn on the central slice in all groups. (**D**) In vivo fluorescence imaging of tumor-bearing mice after injection of Cy-5 labeled DPP and CDPP. The red circles indicate the tumor sites. (**E**) Quantitative fluorescence analysis in the tumor at different times. (**F**) Frozen sections of HeLa tumors after treatment with CDPP or DPP. The tumor vessels and nuclei are stained with FITC-tagged CD31 antibody (green) and DAPI (blue), respectively. Scale bar represents 50 μm. Reprinted with permission from ref [129]. Copyright 2020 Royal Society of Chemistry.

The polycarbonate-based copolymers with various architecture e.g., brushes [120] or graft copolymers [121] were also investigated for GSH-triggered drug delivery systems. However, the most common route to use a GSH-sensitive linkage is to incorporate it into the micelle core as a core-crosslinking agent in order to increase the colloidal stability of micelles in an aqueous medium, improve stability against dilution, avoiding drug leakage, prolonged circulation, and to get redox-sensitivity leading to micelles disintegration followed by the intracellular drug release in tumor cells. Therefore, most research on GSH-responsive nanocarriers focuses on developing core-crosslinked nanoparticles via disulfide bonds. For this reason, the combination of biodegradable polymers with thiol functionalized aliphatic polycarbonates as a hydrophobic part of amphiphilic copolymers are attractive for designing novel biodegradable GSH-responsive nanocarriers. Jing and coworkers described the core-crosslinked PEG-*b*-poly(lactide-*co*-thiol functionalized cyclic carbonate) micelles for GSH-triggered doxorubicin release [122]. In this context, Wang et al. [123] reported a disulfide bond containing biodegradable nanoparticles produced by crosslinking of the partially azidated poly(ethylene glycol)-*b*-poly(ε-caprolactone-*co*-5,5-dibromomethyl trimethylene carbonate) via click chemistry with propargyl 3,3′-dithiopropionate. Subsequently, the authors reported such prepared nanoparticles loaded with paclitaxel and showed the reduction-responsive intracellular drug release, which was verified by confocal laser scanning microscopy studies [124]. Similarly, Zhang et al. [125] reported biodegradable micelles based on poly((ethylene glycol)-*b*-(2,2-dimethyltrimethylene carbonate-*co*-2,2-bis(azidomethyl) trimethylene carbonate)) with a crosslinked core using click chemistry with disulfide containing dialkynyl linker. In this area, Yi et al. [126] also described a highly stable redox-responsive nanocarrier for intracellular doxorubicin delivery, prepared using PEGylated partially crosslinked copolycarbonate obtained by ROP of disulfide-coupled bis-(cyclic carbonate) and trimethylene carbonate monomers using DBU/thiourea derivative (TU) as a catalytic system. In another example, Lu and coworkers synthesized poly((ethylene glycol)-*b*-(5-methyl-5-propargyloxycarbonyl-1,3-dioxane-2-one)) to which 6-bromohexanoic acid and azide-functionalized α-lipoic acid were attached via click reaction. The disulfide core-crosslinked is formed by the addition of a catalytic amount of dithiothreitol [127]. The same group also reported GSH-responsive core-crosslinked biodegradable micelles for doxorubicin delivery into DOX-resistant tumor cells. The nanoparticles with core crosslinked *via* “click” reaction with bis-(azidoethyl)disulfide were made of diblock copolymer: PEGylated propargyl-functionalized APC. In vitro studies of such GSH-sensitive micelles loaded with doxorubicin were conducted using HeLa cells, 4T1 cells, and doxorubicin-resistant ADR/MCF-7 cells which showed a more efficient inhibition of tumor cell growth than the free drug. More importantly, DOX-loaded micelles possessed significantly higher anti-tumor activity against ADR/MCF-7 cells compared to free doxorubicin. It is caused by the “stealth” endocytosis that overcomes the biological barriers of drug-resistant ADR/MCF-7 cells [128]. Overall, such studies highlight that tumor-targeting nanoparticles provide a promising approach to effective drug delivery into multi-drug resistance tumor cells for a large number of clinical chemotherapeutics, which is often a major obstacle to develop an effective tumor therapy. Other limitations of effective chemotherapy with nanoparticles are poor solid tumor penetration by nanoparticles. Very recently, Zhu et al. [129] worked to overcome this limitation by developing a small-sized (~19 nm) redox-responsive nanoparticles based on PEG_2000_-*b*-poly(5-methyl-5-acryloyloxymethylene-1,3-dioxan-2-one), which was cross-linked by Michael addition reaction between pendant acrylate groups and cystamine. Extended in vivo studies using confocal laser scanning microscopy performed with HeLa tumor-bearing mice revealed that DOX-loaded small-sized micelles (DPP) penetrate deeper into tumor parenchyma compared to micelles with an average size ~50 nm (CDPP) (Figure 6). Furthermore, GSH-responsiveness to such small micelles can increase tumor inhibition efficacy due to the rapid release of DOX in highly reducing cytoplasm tumor microenvironment.

In summary, it is evident that redox-responsive nanocarriers based on GSH-triggered disulfide/diselenide bond rupture are capable to efficiently improve the cellular uptake of nanoparticles into tumor tissues, demonstrating their high potential for applications in chemotherapy. However, based on the studies outlined above, redox-sensitive bonding has a dual-use: i.e., triggering the drug release and, equally important, cross-linking of micelles core to enhance their stability and preventing drug leakage. Combining the redox-responsive core cross-linked micelles with another stimulus for drug triggering release could provide a promising platform for cancer treatment.

### 2.3. ROS-Responsive APC Nanocarriers

**Figure 7:**
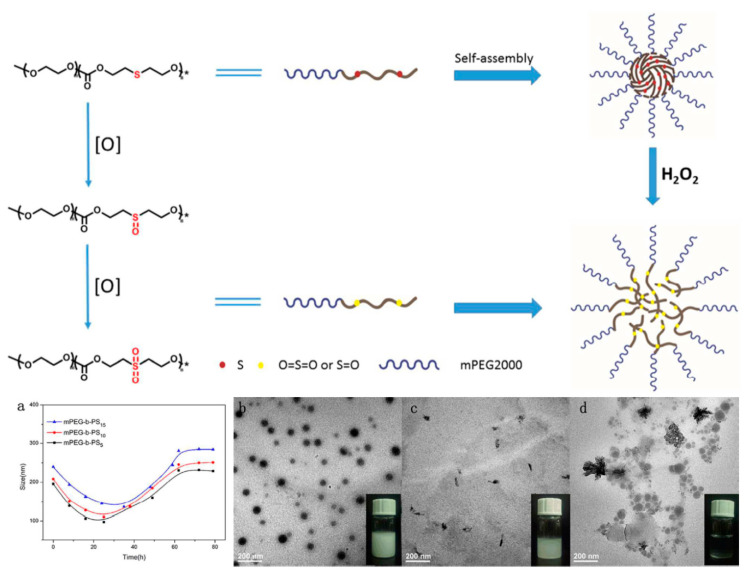
(**Top**) Schematic illustration of self-assembly of the PEGylated thioether-containing APC and its oxidation behaviors. (**Bottom**) The micelles size changes determined by DLS (a) and corresponding TEM images (b–d) at different oxidation times (0, 30 and 60 h, respectively) in the presence of H_2_O_2_ at 37 °C. Reprinted with permission from ref [134]. Copyright 2018 Royal Society of Chemistry.

There are a lot of studies pointing that tumor tissues are characterized by a higher level of reactive oxygen species (ROS) compared to healthy cells. Endogenous cancer cells constantly produce ROS (e.g., hydroxyl radical (·OH), hydrogen peroxide (H_2_O_2_), superoxide (O_2_^−^), singlet oxygen (^1^O_2_), etc.) as the byproducts of aerobic metabolism caused by oncogenic transformation, intensive metabolism related to increased proliferation, or mutations in mitochondrial DNA [130]. A high level of ROS in tumor tissues may cause a variety of physiological responses, such as the formation of DNA mutations and genetic instability, cell adaptation, or increased proliferation rate [131]. The phenomenon of a higher level of ROS in cancer cells prompted scientists to develop ROS-triggered nanocarriers to release anticancer drugs in response to elevated ROS concentration in tumor site-specific regions. In this regard, some studies suggest that the ROS-responsive nanocarriers family has great potential for cancer treatment and inflammation targeting [132,133]. However, the mechanism of the oxidation process and the safety of oxidation byproducts have to be evaluated before clinical use. Yan et al. [134] recently reported biodegradable oxidation-responsive nanocarriers. Respectively, poly((ethylene glycol)-*b*-(carbonate-thioether)) was synthesized by lipase-catalyzed ROP of the cyclic diethylene sulfide carbonate dimer. The thioether groups located in the copolymer main chain caused the nanocarriers to exhibit ROS-sensitivity by oxidizing the thioether groups to hydrophilic polar sulfoxides or sulfones. This causes the size of the micelles to decrease, which was attributed to the reduction in the core of the micelles. Further oxidation increased the hydrophilicity of the copolymer and led to an increase in the size of the micelles, which was caused by the formation of loose aggregates (Figure 7). MTT assays showed that the thioether-containing APC and their oxidized products were non-toxic. Furthermore, the authors paid particular attention to clarify the mechanism of the oxidation process to provide a promising platform for cancer treatment. 

Wang et al. [135] developed ROS-responsive core crosslinked APC nanocarriers for the delivery of doxorubicin. The PEGylated APC with a pendant alkynyl group was coupled with thioketal containing azide derivative (bis(2-azidoethyl)-3,3′-(propane-2,2-diylbis(sulfanediyl))dipropanoate) via “click” reaction. The introduction of thioketal containing core-crosslinker endowed ROS-responsiveness for triggered intracellular anticancer drug release and improved stability of nanocarrier. The DOX-loaded core-crosslinked micelles with the thioketal group show low biotoxicity and had significantly higher toxicity effects for cancer cells (HeLa and MCF-7) compared to both the non-crosslinked micelles and non-responsive cross-linked micelles. Moreover, CLSM studies revealed an internalization efficiency and drug release inside the cancer cells. Yang et al. [136] developed ROS-responsive nanocarriers based on selenium-containing amphiphilic APC. The DOX-loaded nanoparticles were rapidly disrupted under biologically relevant concentrations of H_2_O_2_, thus releasing the encapsulated drug for inducing cancer cells apoptosis. Moreover, in vitro cytotoxicity studies revealed that these DOX-loaded nanoparticles inhibit the proliferation of cancer cells while exhibiting much lower cytotoxicity in healthy cells. Yu et al. [137] designed and synthesized a series of ethyl selenide, phenyl selenide, or ethyl telluride groups-functionalized APC for ROS-responsive photodynamic therapy. The PEGylated chalcogen-containing APC nanoparticles rapidly disintegrate under triggering in ROS conditions releasing encapsulated drugs. The oxidation mechanism and kinetics studies revealed that telluride-functionalized nanoparticles degraded much faster than selenides-containing ones. To evaluate the ROS-triggered drug release, DOX and photosensitizer chlorin e6 (Ce6) were loaded. Upon red light irradiation, Ce6 generates ^1^O_2_ that triggers the degradation of the nanocarrier. It results in an acceleration of DOX release to achieve efficiently the combination of photodynamic therapy and chemotherapy. In view of the potential of clinical application of ROS-responsive APC nanocarriers, the same group expanded their research in this field. More recently, Yu et al. [138] developed theranostic nanocarriers for delivering anticancer drugs to the ROS-overproduced tumor tissues and fluorescent monitoring of the intracellular redox status simultaneously. Nanocarriers were prepared from the triblock copolymer consisting of PEG and two APC blocks, functionalized with ethyl selenide and coumarin-based chromophore respectively. The nanoparticles formed can be loaded with two anticancer drugs—PTX through hydrophobic interactions and cis-platin through coordination. Moreover, dual-drug loaded nanocarriers showed increased stability against dilution due to additional cross-linking through Se-Pt coordination. Nanocarriers loaded with two anticancer agents selectively kill triple-negative breast cancer cells and show reduced toxicity to the healthy cells. So far, there are only a few reports that present the use of ROS-responsive nanocarriers based on aliphatic polycarbonates for anticancer drug delivery. Further research in this field may thus provide new possibilities for tumor treatment.

### 2.4. Light-Responsive APC Nanocarriers

**Figure 8:**
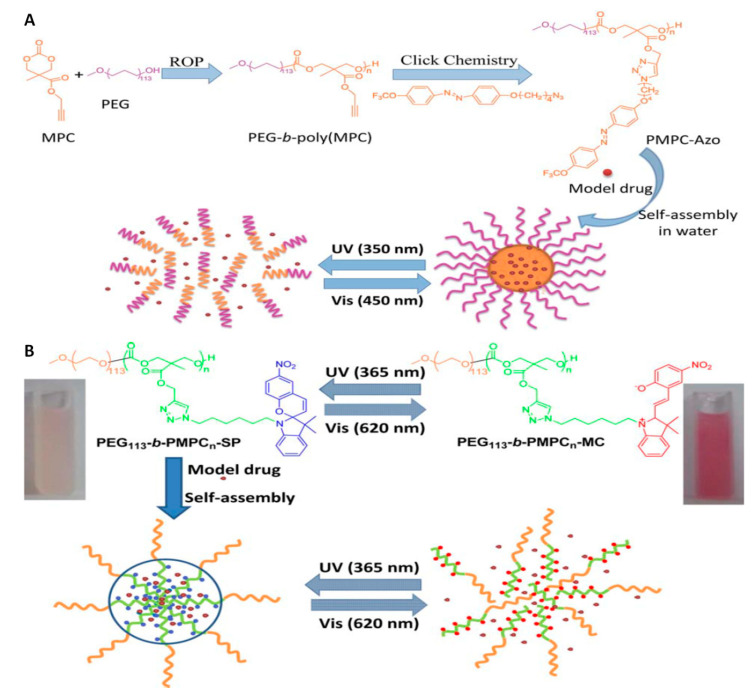
(**A**) Synthesis of PEGylated trifluoromethoxy-azobenzene-decorated poly(carbonate)s and schematic illustration of reversible light-responsive micelles for drug packaging and release. Reprinted with permission from ref [143]. Copyright 2014 Royal Society of Chemistry. (**B**) Schematic illustration of micelle assembly and disassembly of photo-responsive amphiphilic APC containing spiropyran group. Reprinted with permission from ref [144]. Copyright 2014 Wiley Periodicals, Inc.

The convenient and clean nature of light made the photoactivated nanocarriers an extensively studied drug delivery systems. What distinguishes light-responsive nanocarriers from other stimuli-responsive carriers is their ability to achieve a precise on-demand drug release in a spatiotemporal manner in response to non-invasive light irradiation with a specific wavelength [139]. The phototoxicity of high energy irradiation (X-rays, γ-rays) is clinically used in radiotherapy whereby damaging the DNA of tumor tissues leading to cellular death [140]. However, high-energy radiation damages healthy tissues, which precludes the use of this type of radiation as a stimulus. The light used as a trigger to release drugs from nanocarriers must be of lower energy. Therefore, light-responsive nanocarriers are mostly based on UV, VIS, or NIR irradiation [141]. An additional advantage of light-triggered drug delivery systems is the low toxicity of photosensitive groups, which can be incorporated into the main copolymer chain or functionalized as a side group. The response of the photosensitive group may be irreversible or reversible, however, it is mainly based on the processes of photoisomerization, photoreduction, photolysis, change of electrostatic charge and lead to the release of the therapeutic agent [142]. Lu and coworkers designed photosensitive APC micelles by introducing a trifluoromethoxy-azobenzene as a side group via “click” chemistry [143]. Azobenzene group undergo trans–cis photoisomerization in response to UV and VIS light. The *cis* form of the azobenzene group is more polar than the trans. The isomerization of trans-azobenzene group functionalized APC micelles into the cis-azobenzene group under 365 nm UV irradiation caused a micelles disintegration with the simultaneous release of the encapsulated drug. It was caused by a disturbance of the hydrophilic-hydrophobic balance. Subsequent irradiation of copolymer with the visible light (450 nm) caused micelles to form once again. This phenomenon occurred due to the azobenzene cis-trans reverse isomerization (Figure 8A). 

**Figure 9:**
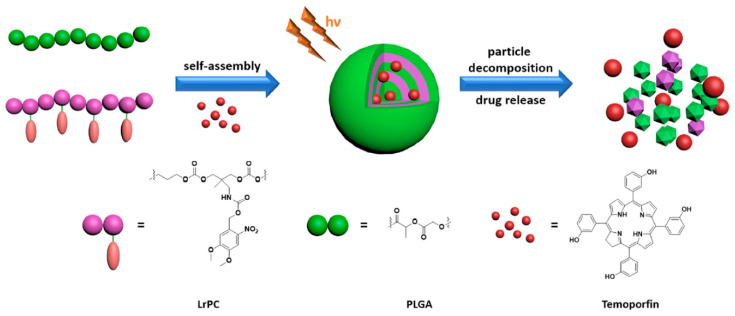
Schematic illustration of the self-assembly of temoporfin-loaded nanoparticles from light-responsive APC and poly(lactide-*co*-glycolide) and UV light-responsive drug release. Reprinted with permission from ref [147]. Copyright 2018 American Chemical Society.

In this context, the same group reported light-sensitive micelles consisting of PEGylated APC functionalized with spiropyran chromophore group in the side chain [144]. The photosensitivity of this system was caused by the conversion of hydrophobic spiropyran into hydrophilic merocyanine under 365 nm UV light irradiation. As a result, the disintegration of micelles and the subsequent release of the encapsulated hydrophobic drug is observed. Exposing the micelles to visible light (620 nm), the aggregation process was observed again with subsequent re-encapsulation of drugs (Figure 8B). These works provide a smart and convenient approach to design smart vehicles for the release of various hydrophobic molecules. Lu and coworkers have continued to develop novel light-responsive APC nanocarriers. PEGylated APC with pendent *o*-nitrobenzyl ester group was reported [145]. The photo-cleavage nature of the *o*-nitrobenzyl ester group into the free carboxylic acid group and *o*-nitrosobenzaldehyde under UV light irradiation caused micelles disassembly and the release of the encapsulated drug. Similarly, Fang et al. [146] reported photo-responsive micelles consisting of PNIPAM-*b*-poly(3-methyl-3-nitrobenzyl-trimethylene carbonate) for indomethacin drug delivery system. Likewise, Kuckilng and coworkers developed a series of light-degradable nanocarriers consisting of PEGylated APC with an *o*-nitrobenzyl ester group as side chains [147,148,149,150,151]. The nanoparticles were prepared from mixing amphiphilic APC copolymer with poly(lactide-*co*-glycolide) in ratio 1:3 (Figure 9). The nanoparticles were stable in dark, however, the light-triggered cleavage of the *o*-nitrobenzyl ester groups led to degradation of the copolymers via intramolecular cyclization into small molecules. The photoactivated degradation of nanocarriers results in a burst release of the encapsulated drug (temoporfin). Preliminary in vitro cellular studies of the above-mentioned nanoparticles and degradation products confirmed that systems have the potential to be used for in vivo studies. 

Very recently, the same group reported light-degradable APCs nanoparticles by introducing bromocoumarin groups into the side chain [152]. Upon light irradiation, the photo-degradable bromocoumarin side groups are removed, which causes the appearance of nucleophilic amino groups. As a result, the copolymer undergoes amine induced degradation into low molecular weight compounds. In addition, the authors suggest that the bromocoumarin group, compared to the *o*-nitrobenzyl one, shows better biocompatibility, does not form toxic degradation products and characterizes a higher photolysis rate. Based on this strategy, a variety of photoactivated nanoparticles can be obtained to expand the family of aliphatic polycarbonates nanocarriers as highly controlled spatially and temporally drug delivery systems. Overall, light-sensitive nanocarriers represent a promising option for controlled drug delivery systems. Although the endogenous stimuli-responsive nanocarrier approach is limited due to the heterogeneity of the tumor tissues, the complexity of the light-triggered drug release mechanism and the potential toxicity of the photosensitive groups and their degradation products are still major disadvantages of photosensitive systems. However, the simultaneous combination of endogenous and exogenous stimulus-responsive groups into a biodegradable nanocarrier may provide more precise cancer treatment with reduced side effects.

### 2.5. Dual/Multiple Stimuli-Responsive APC Nanocarriers

The rapid and uncontrolled growth of cancer cells is associated with the unique characteristic tumor microenvironment. As cancer develops, tumors become more and more heterogeneous [153]. Thereby, the levels of characteristic pathophysiological markers (i.e., pH, GSH, ROS, enzyme level or various vitamin receptors overexpression, etc.) differ from cell to cell. Therefore, it would be highly advantageous to use combinations of two or more stimuli to further enhance the tumor-targeted effect, response rate or achieve on-demand burst drug release in tumor site-specific regions. Multi-stimuli-sensitive nanocarriers have so far shows much more advantages over single-stimuli responsive ones. Herein, we emphasize the development of dual- or multi-stimuli-responsive nanocarriers consisting of aliphatic polycarbonates.

Recently, most research has focused on combining pH sensitivity with various endogenous stimuli, e.g., redox, ROS. The combination of pH and redox-sensitivity is attractive because tumor tissues are always characterized by low pH and elevated level of intracellular GSH. Taking advantage of this, Zhong and coworkers designed and developed a pH/GSH-responsive micelles consisted of diblock copolymer poly(ethylene glycol)-*b*-(disulfide bond)-poly(2,4,6-trimethoxybenzylidene pentaerythritol carbonate) for dually-triggered intracellular release of doxorubicin [154]. The acetal bonds located in the core-forming block of micelles hydrolyzes in endosomes causing nanocarriers to swell leading to partial drug release. Following the movement of micelles from the endosomes to the cytosol, disulfide bonds are cleaved due to the high concentration of GSH, leading to the rapid release of the encapsulated drug. In vitro, drug release studies revealed that dual pH- and redox-sensitive micelles exhibit synergistic drug release effects compared with pH-responsive. Hydrolysis of the acetal groups to two hydroxyl groups increases the hydrophilicity of the micelle core, which significantly facilitates the reduction-triggered DOX release. The sensitivity to two stimuli also explains better anticancer activity compared with only pH-responsive nanocarriers. Similarly, Yi et al. [155] reported nanoparticles prepared from the poly((ethylene glycol)-*b*-(2,4,6-trimethoxybenzylidene- pentaerythritol carbonate-*co*-5-methyl-5-propargyl-1,3-dioxan-2-one)) obtained by organocatalyzed ROP. The propargyl groups were used to cross-link the core via “click” reaction with 1,6-diazidohexane or bis(azidoethyl) disulfide to obtain insensitive and GSH-sensitive crosslinking, respectively. Yang and coworkers designed and prepared smart pH/redox dual-responsive micelles with high drug loading for anticancer drug delivery [156] and reported micelles consisted of PEGylated polycarbonate that contains disulfide-functionalized carboxylic acid groups (PEG-*b*-(APC-SS-COOH)). Interestingly, the effect of hydrophilic/hydrophobic block lengths and drug loading efficiency was studied. The DOX loading capacity increased with an increasing ratio of the carboxylic acid groups in the copolymer side chain. In vitro drug release showed that at the endolysosomal pH (~5.0) and in the presence of GSH at the cytoplasmic concentration (10 mM GSH), DOX release was significantly accelerated. Moreover, these DOX-loaded dual-sensitive nanocarriers accumulate in tumor tissues, as shown by confocal microscopy studies, and demonstrated better inhibition of tumor growth in nude mice bearing BT-474 xenografts. In this context, Hu et al. [157] developed dual-responsive nanovesicles by introducing disulfide and tertiary amine groups into the APC copolymer backbone. 

Another research reported dual-responsive micelles obtained through mixing positively charged pH/reduction-sensitive APC copolymer possessed hydrazone linked doxorubicin and disulfide functional pendant groups, with negatively charged PEGylated APC copolymer containing acid-labile β-carboxylic amide as side groups [158]. The hydrazone-bond linked DOX was effectively released under endosomal pH and 10 mM GSH conditions. The confocal microscopy studies and MTT assays conducted on HeLa cells showed enhanced intracellular uptake efficacy and better antiproliferative properties compared with free DOX. 

**Figure 10:**
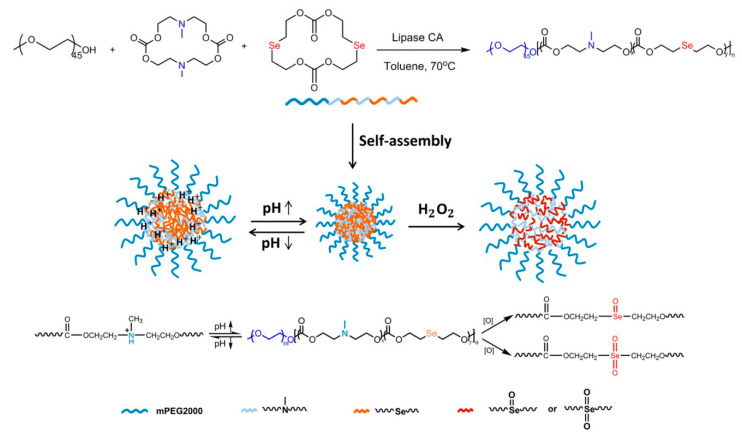
(**Top**) Synthesis of PEGylated APC containing selenide and tertiary amine groups in the polymer backbone by lipase-catalyzed ring-opening copolymerization. (**Bottom**) Schematic illustration of dual-responsive nanocarriers for ROS/pH-controlled drug release. Reprinted with permission from ref [160]. Copyright 2019 Royal Society of Chemistry.

Dual-responsiveness to pH and ROS is also an attractive modification of nanocarriers for targeted tumor chemotherapy, because cancer cells display low pH and elevated ROS level. Yang and coworkers designed pH/ROS-sensitive nanocarriers based on a mixture of two diblock copolymers (i) PEGylated carboxylic acid-functionalized thioether-containing APC and (ii) PEGylated phenylurea-functionalized thioether-containing APC [159]. The addition of the PEGylated copolymer containing phenylurea groups enhanced the colloidal stability of nanocarriers. Whereas, oxidation of the thioethers group to sulfoxide followed by the subsequent oxidation of the later to sulfone group causes an increase in hydrophilicity in the hydrophobic core of micelles. The micelles were highly sensitive to pH and ROS as shown in the in vitro DOX release studies. In vivo studies revealed that dual-responsive micelles accumulated in the tumor tissues upon the enhanced permeability and retention effect in a PC-3 xenograft mouse model. The nanocarrier consisting of poly(ethylene glycol)-*b*-polycarbonate with incorporated selenide and tertiary amine groups into the APC backbone (Figure 10) was also reported to be pH/ROS-dual-responsive platform for cancer chemotherapy [160]. 

**Figure 11:**
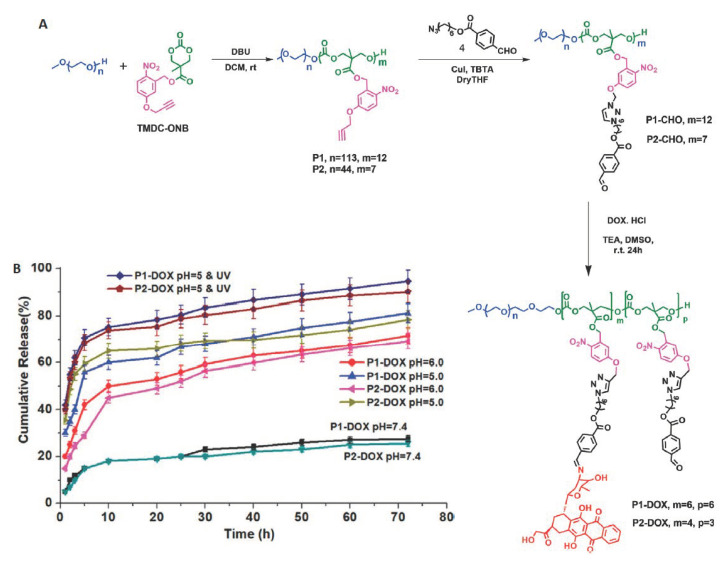
(**A**) Synthesis of photo- and pH-responsive poly(ethylene glycol)-poly(carbonate) block copolymer with nitrobenzyl ester and doxorubicin molecules conjugated via acid-sensitive Schiff-base linkage side groups. (**B**) In vitro cumulative release of DOX at pH 7.4, 6.0, 5.0, and pH 5.0 with 10 min UV light irradiation. Reprinted with permission from ref [164]. Copyright 2020 WILEY-VCH Verlag GmbH and Co. KGaA, Weinheim, Germany.

Yu and coworkers reported fluorophore-installed APC nanocarriers possessing incorporated selenide and tertiary amine groups in the copolymer backbone [161]. Among the various dual-responsive nanocarriers, pH/light-responsive systems have been also reported. Lu and coworkers designed photo/pH-responsive nanocarriers for anticancer drug release based on a triblock amphiphilic aliphatic polycarbonate containing azobenzene and 2-azido-1-ethyl-diethylamine groups as side chains [162]. Then the same authors designed triple-stimuli-responsive micelles which respond to the changes in GSH, light and temperature. APC-based micelles consisted of a thermo-sensitive shell by introducing tetraethylene glycol as a pendant group and photo-responsive poly(2-nitrobenzyl methacrylate) which was linked via GSH-sensitive disulfide bond. The use of a triple stimulus allowed control of the changes in the morphology of nanoparticles, and thus for a precise, controlled release of drugs [163]. Kalva et al. [164] reported pH/photo-dual-sensitive nanocarriers. The micelles consisted of PEGylated APC bearing photo-responsive *o*-nitrobenzyl ester group and doxorubicin molecules conjugated via acid-sensitive Schiff-base linkage. Interestingly, both triggers can be used individually or together for adjusting the drug release rate (Figure 11). 

Very recently, Wang et al. [165] reported GSH/ROS-sensitive nanoparticles consisting of PEGylated poly(ester-*co*-carbonate) obtained via enzymatic copolymerization of ε-caprolactone and diselenic carbonate macrolide. A detailed study of the copolymerization made it possible to obtain a copolymer with a controlled number of diselenide groups in the main chain to ensure a highly controlled GSH/ROS-responsive drug release from the nanoparticles. Sun et al. [166] designed and developed pH/GSH/ROS-triple sensitive nanovehicles for activated intracellular DOX release. The reported micelles were formed from PEGylated APC copolymer containing diselenide and tertiary amine groups in the backbone. Both bioresponsive groups allowed for pH/GSH/ROS-triple responsiveness, resulting in a highly controlled drug release. The confocal microscopy studies indicated facilitated cellular uptake and intracellular DOX release. The heterogeneity and complexity of the tumor tissues make it extremely difficult to achieve precise and highly controlled drug release in tumor site-specific regions. In addition, levels of pathophysiological markers are highly patient-dependent and vary with disease states. However, it seems that the application of two or more stimuli with synergistic action may increase efficient drug delivery. Therefore, personalized therapy with nanocarriers sensitive to two or more stimuli, depending on the type of cancer and its condition, should be considered in order to ensure the best therapeutic effect.

## 3. Conclusions

The introduction of Doxil^®^ as an anticancer agent has shown how powerful a weapon the use of nanoparticles can be in the fight against cancer. Furthermore, it is also worth noting that an analysis of the American Food and Drug Administration and European Medicines Agency databases indicates that more and more novel nanocarriers are approved for the diagnosis and treatment of cancer, including systems using active targeting or stimulus-responsivity mechanisms [167]. This prompts scientists to develop more efficient systems to tailor individual therapy that targets cancer cells and circumvents multi-drug resistance. Excellent biodegradability and biocompatibility, combined with numerous modification possibilities in the monomer and copolymer structure to achieve superior tumor-targeting efficiency, makes nanocarriers based on aliphatic polycarbonates ideal candidates for anticancer drug delivery systems. Therefore, the use of APC as stimuli-responsive nanocarriers has been extensively studied in the past decade. Some efforts have focused on the design of nanocarriers responsive to endogenous stimuli e.g., pH, GSH, ROS, or the overexpression of various vitamin receptors. Considering that the ability of such systems to release anticancer drugs with such small changes in pH, ROS or GSH is not an easy task, even if the risk-benefit ratio is definitely favorable for nanocarriers, it should be assessed before clinical use. The use of an exogenous stimulus such as light also appears to be attractive because of the possibility of achieving a controlled on-demand release of a drug in a spatiotemporal manner. To provide a better therapeutic outcome and tumor-targeting efficiency a versatility of dual/multi-responsive nanocarriers based on APC has also been studied. The use of a nanocarrier sensitive to two or more stimuli allows the ability to target the tumor cells with a very precisely controlled drug release manner in tumor tissues. Besides, the encapsulation of fragile chemotherapeutic agents in nanocarriers significantly improves their efficacy by avoiding systemic side effects and reducing the doses of drugs administered. In spite of all this, most of the multi-stimuli-responsive systems are sophisticated in design which makes clinical development more complex. In particular, the mechanism and benefits of each stimulus used should be assessed. In addition, recent attention is given to the ligand-decorated nanocarriers which can specifically recognize and target the tumor tissues. The ligand-installed nanocarriers showed an enhanced internalization by tumor tissues, improved cellular uptake of the encapsulated drugs, and reducing systemic side effects which were excellently described by Kataoka and coworkers [168]. In the future, the combination of stimuli-responsive biodegradable nanocarriers with the addition of active targeting ligands to nanoparticle surfaces might mitigate some of the challenges in chemotherapy. 

In the fight against cancer, current advances in research on stimulus-responsive systems are a promising strategy with a bright future. The successful clinical implementation of stimuli-responsive nanocarriers based on aliphatic polycarbonates remains a major challenge, even though recent results are promising. In future studies, there is an expectation of more efficient nanocarriers with a superior balance between safety, tumor-targeting ability, and clinical outcome.
